# Ethylene and *RIPENING INHIBITOR* Modulate Expression of *SlHSP17.7A, B* Class I Small Heat Shock Protein Genes During Tomato Fruit Ripening

**DOI:** 10.3389/fpls.2020.00975

**Published:** 2020-06-30

**Authors:** Rakesh K. Upadhyay, Mark L. Tucker, Autar K. Mattoo

**Affiliations:** ^1^ Sustainable Agricultural Systems Laboratory, The Henry A. Wallace Beltsville Agricultural Research Center, United States Department of Agriculture-ARS, Beltsville, MD, United States; ^2^ Soybean Genomics and Improvement Laboratory, The Henry A. Wallace Beltsville Agricultural Research Center, United States Department of Agriculture-ARS, Beltsville, MD, United States

**Keywords:** gene expression, ethylene, 1-MCP, *SlMADS-RIN*, *small heat shock protein genes*, tomato, tomato ripening mutants

## Abstract

Heat shock proteins (HSPs) are ubiquitous and highly conserved in nature. Heat stress upregulates their gene expression and now it is known that they are also developmentally regulated. We have studied regulation of small HSP genes during ripening of tomato fruit. In this study, we identify two small HSP genes, *SlHSP17.7A* and *SlHSP17.7B*, localized on tomato Chr.6 and Chr.9, respectively. Each gene encodes proteins constituting 154 amino acids and has characteristic domains as in other sHSP genes. We found that *SlHSP17.7A* and *SlHSP17.7B* gene expression is low in the vegetative tissues as compared to that in the fruit. These sHSP genes are characteristically expressed in a fruit-ripening fashion, being upregulated during the ripening transition of mature green to breaker stage. Their expression patterns mirror that of the rate-limiting ethylene biosynthesis gene ACC (1-aminocyclopropane-1-carboxylic acid) synthase, *SlACS2,* and its regulator *SlMADS-RIN*. Exogenous application of ethylene to either mature green tomato fruit or tomato leaves suppressed the expression of both the *SlHSP17.7A, B* genes. Notably and characteristically, a transgenic tomato line silenced for *SlACS2* gene and whose fruits produce ~50% less ethylene *in vivo*, had higher expression of both the *sHSP* genes at the fruit ripening transition stages [breaker (BR) and BR+3] than the control fruit. Moreover, differential gene expression of *SlHSP17.7A versus SlHSP17.7B* gene was apparent in the tomato ripening mutants—*rin/rin*, *nor/nor*, and *Nr/Nr*, with the expression of *SlHSP17.7A* being significantly reduced but that of *SlHSP17.7B* significantly upregulated as compared to the wild type (WT). These data indicate that ethylene negatively regulates transcriptional abundance of both these sHSPs. Transient overexpression of the ripening regulator *SlMADS-RIN* in WT and ACS2-AS mature green tomato fruits suppressed the expression of *SlHSP17.7A* but not that of *SlHSP17.7B*. Thus, ethylene directly or in tune with *SlMADS-RIN* regulates the transcript abundance of both these sHSP genes.

## Introduction

Heat shock proteins (HSPs) are ubiquitous in nature and highly conserved in living organisms (Plesofsky-Vig et al., 1992; Waters and Vierling, 1999; Kappé et al., 2002; Franck et al., 2004; Fu et al., 2006; Aevermann and Waters, 2008; Waters, 2013). They are prominent in cells/tissues exposed to elevated temperatures (Sun et al., 2002; Waters, 2013; Zhang et al., 2015) as well as to chilling temperatures (Ré et al., 2016). HSPs are classified based on their molecular weight, namely, HSP100s, HSP90s, HSP70s, HSP60s, HSP20s and small HSPs (sHSPs) (Waters, 2013). sHSPs constitute low molecular weight proteins that function as molecular chaperones critical for protein folding and prevention of irreversible protein aggregation (Becker and Craig, 1994; Hartl, 1996; Liberek et al., 2008; Giorno et al., 2010; Tyedmers et al., 2010; Waters, 2013). In addition to sHSPs being prominently expressed during heat shock response in plants, it is now known that some are expressed in unstressed cells as well and are, therefore, involved in processes other than heat stress (Tyedmers et al., 2010; Waters, 2013). For example, in plants, they are upregulated during ripening initiation of tomato fruit (Goyal et al., 2012; Shukla et al., 2017) and may also protect ripe tomato fruits against chilling injury (Ré et al., 2016).

Ethylene regulates plant processes such as fruit ripening independently as well as in conjunction with other hormones and molecules (Fluhr and Mattoo, 1996; Giovannoni et al., 2017; Mattoo and Upadhyay, 2019). Ethylene directly or indirectly promotes transcription/translation of numerous ripening-related genes, including those associated with cell wall breakdown, carotenoid biosynthesis, aroma development, pigment accumulation, fruit softening, and flavor (Gray et al., 1994; Barry and Giovannoni, 2007; Klee and Giovannoni, 2011). Tomato is one of the models for dissection of ethylene-mediated regulation of genes during fruit ripening, facilitated by the availability of nonripening tomato lines, such as *ripening-inhibitor (rin/rin)*, *nonripening (nor/nor)*, and *never-ripe (Nr/Nr),* in which ethylene production is compromised (Mattoo and Vickery, 1977; Oeller et al., 1991; Picton et al., 1993; Wilkinson et al., 1995; Hackett et al., 2000; Alexander and Grierson, 2002; Moore et al., 2002; Vrebalov, 2002; Giovannoni, 2007; Razdan and Mattoo, 2007). Of these mutants, *ripening-inhibitor* (*rin*) mutation encodes a MADS-box transcription factor *SlMADS-RIN* that regulates genes involved in fruit ripening (Ng and Yanofsky, 2001; Vrebalov, 2002; Hileman et al., 2006; Martel et al., 2011; Fujisawa et al., 2013).

Tomato genome harbors five members of class I sHSP genes (Goyal et al., 2012; Yu et al., 2016; Shukla et al., 2017). The involvement of sHSPs in fruit biology became apparent by earlier studies which demonstrated *VISCOSITY 1* (*VIS1*) as a regulator of pectin depolymerization affecting juice viscosity of tomato fruit (Ramakrishna et al., 2003) while *HSP21* was shown to stabilize photosystem II of photosynthesis against oxidative stress in addition to promoting color change during tomato fruit ripening (Neta-Sharir et al., 2005). More recently, a unique intron-less cluster of three sHSP chaperone genes, *SlHSP17.6*, *SlHSP20.0* and *SlHSP20.1*, was shown to be resident on the short arm of chromosome 6 and found differentially expressed during tomato fruit ripening (Goyal et al., 2012). Further, it was shown that ethylene suppresses the transcription of the latter tomato sHSP gene cluster during the transition of mature green stage to ripening initiation and involves SlMADS-RIN box protein (Shukla et al., 2017).

Here we identify, characterize and present transcriptional regulation of two novel duplicated members of class I sHSPs in tomato, *SlHSP17.7A* and *SlHSP17.7B*, during fruit ripening. Further, expression of *SlHSP17.7A* gene was found minimal in the isogenic ripening mutants of Alisa Craig—*rin/rin*, *nor/nor*, and *Nr/Nr*, while that of *SlHSP17.7B* was higher in these mutants. We also utilized an ethylene-deficient tomato line to demonstrate that ethylene biosynthesis directly and/or indirectly regulates the expression of sHSP genes in tomato.

## Materials and Methods

### Plant Materials, Transgenic Tomato Lines, and Sample Collection

Wild-type tomato (*Solanum lycopersicum* cv. Ailsa Craig) and its near isogenic mutant lines—*ripening-inhibitor (rin/rin)*, *nonripening (nor/nor)* and *never-ripe (Nr/Nr)*—and previously characterized ACC synthase 2 (*ACS2*)-silenced transgenic line (ACS2-AS) together with its azygous/wild-type control tomato (WT-Ohio8245) (Mehta et al., 2002; Sobolev et al., 2014) were employed for the studies presented here. sHSP gene expression was quantified at distinct ripening stages: mature green (-5BR), breaker (BR), pink (breaker+3), and red ripe (breaker+7). All these genotypes were grown in a temperature-controlled greenhouse under natural light conditions. Vegetative tissues, leaf, stem, flower, and roots, were collected from 1-month-old wild-type Ailsa Craig tomato plants. *For seedling collection*, 8- to 10-day-old 50-germinated seeds in triplicates with two cotyledons were immediately frozen in liquid nitrogen and stored at −80°C until used. *For development studies*, fruits tagged at anthesis (DPA: days post anthesis) were collected at 8 DPA (developmental stage 1), 15 DPA (developmental stage 2), and 22 DPA (developmental stage 3). *For ripening studies*, tomato fruits at 5 days before breaker (-)5BR—equivalent to mature green (MG) stage, breaker (BR) and red ripe stage [8 days after breaker (BR+8)] were collected. Fruits from the WT-Ohio8245 were harvested at (-)5BR, (MG), BR, and BR+8 stages. Pericarp tissue excised from harvested fruits was immediately frozen in liquid nitrogen and stored at −80°C until used (Mehta et al., 2002). A minimum of 3 biological replicates were used for each experiment.

### Identification, Sequence Alignment and Phylogenetic Analysis of SlHSP17.7A and SlHSP17.7B

Two novel ripening-specific genes *SlHSP17.7A* (NM_001279116.2/Solyc06g076520.1) and *SlHSP17.7B* (XM_015231817.1/Solyc09g015020.1) were identified by utilizing prior information on the following genes: *SlHSP17.6* (AY150039/Solyc06g076540.1.1), *SlHSP20.0* (AJ225048/Solyc06g076570.1.1), and *SlHSP20.1* (AJ225046/Solyc06g076560.1.1) (Goyal et al., 2012; Shukla et al., 2017). The latter tomato HSPs were used as query sequences for BLAST P search in gene bank (https://blast.ncbi.nlm.nih.gov) as well as with BLAST P program in tomato genome [International Tomato Genome Sequencing Consortium (SGN; solgenomics.net) database, version ITAG 2.4] to look for similar sequences. ExPASy bioinformatics resource (https://www.expasy.org) portal was used for the prediction of molecular weight and isoelectric point (PI). Individual domains in the protein sequences were identified and manually highlighted. Multiple sequence alignment was performed using MUSCLE program (http://www.ebi.ac.uk/Tools/msa) and primers were designed using Primer3 program (http://bioinfo.ut.ee/primer3-0.4.0/). Forty-two similar sequences were extracted from tomato genome database to generate the phylogeny among tomato sHSPs (Goyal et al., 2012; Paul et al., 2016; Yu et al., 2016; Shukla et al., 2017; **Supplementary Table 1**). The phylogenetic tree was constructed by maximum likelihood method based on JTT matrix model with 1000 boot strap values using Mega 7 program (Kumar et al., 2016). The analysis involved 42 amino acid sequences. All positions with less than 95% site coverage were eliminated. For qRT-PCR analysis, forward primer was made from 3′ coding DNA sequence while reverse primer was designed from 3′ UTR due to sequence degeneracy within the five closely related class I tomato HSP members. Primer sequences used in qRT-PCR along with their gene bank accessions and SGN identity numbers are listed in **Supplementary Table 2**.

### Exogenous Ethylene and 1-Methylcyclopropene Treatments

Mature green [MG/(-)5BR] tomato fruits from WT-Ohio8245 control and ACS2-AS transgenic lines were treated with 25 ppm ethylene or 2 ppm 1-Methylcyclopropene (1-MCP) (AgroFresh, Goyal et al., 2012 Collegeville, PA, USA) in triplicate from three independent biological replicates (Shukla et al., 2017). A third set of fruits left in the open air was considered as a control for the experiment. Treated and control fruit were collected at 0, 12, and 24 h of treatment. Ethylene (25 ppm) treatment of 8–10 fully expanded leaves from mature WT-Ailsa Craig plants was carried out for 0, 24, 48, 72, and 96 h in the dark at 25°C. Samples were removed at the indicated time points and immediately flash frozen at −80°C until used.

### Total RNA Extraction, cDNA Synthesis, and Quantitative Real-Time PCR (qRT-PCR)

Total RNA was extracted from 100 mg of each sample using Plant RNeasy kit according to manufacturer’s instructions (Qiagen). Isolated RNA was first subjected to RNase-free DNase (Qiagen) treatment to eliminate genomic DNA contamination and then purified using a RNeasy Mini Kit (Qiagen). RNA samples with an A*_260/280_* ratio of 1.8–2 were electrophoresed on agarose gels to ensure the presence of intact rRNA bands (Goyal et al., 2012). Methods for cDNA synthesis and qRT-PCR were essentially as described previously (Bustin et al., 2009; Shukla et al., 2017; Upadhyay et al., 2017). Relative gene expression was quantified according to 2^−ΔΔCT^ method (Livak and Schmittgen, 2001). Tomato genes *SlTIP41* and *SlUBI3* were used as standard housekeeping genes to normalize the expression of target genes (Expósito-Rodríguez et al., 2008; Mascia et al., 2010). For relative expression data, the threshold cycle (C_T_) values for all the genes of interest (C_T_ of GOI) were normalized to the geometric mean C_T_ value obtained from the two tomato reference genes (C_T_ tom Refs) as ΔCT = (CT of GOI) − (geometric mean CT of *SlTIP41* and *SlUBI3*) (Vandesompele et al., 2002). For fruit ripening studies, mature green/(-)5BR was taken as a calibrator to calculate the 2^−ΔΔCT^ for relative gene expression values. Primer efficiency was calculated for each primer pair as: efficiency=10^(-1/slope). Primer pairs with primer efficiency above 90% were used for the study. qRT-PCR data represent average ± standard deviation from a minimum of three independent biological replicates for each gene.

### *In Silico* Analysis of *SlHSP17.7A* and *SlHSP17.7B* Promoters for Predicting *cis*-Elements

International Tomato Genome Sequencing Consortium (SGN; solgenomics.net) database (version ITAG 2.4) was used to extract promoter region sequences (≈ 2 kb of the 5′ upstream region of the start codon) of *SlHSP17.7A* and *SlHSP17.7B* genes. Plant CARE relational database (Lescot et al., 2002) and PLACE (the plant-*cis*-acting regulatory DNA elements) database (Higo et al., 1999) were used for plant *cis-*element searches in both the gene promoters (**Supplementary Tables 3** and **4**).

### Overexpression, Construct Preparation, and Agro-Injection of Tomato Fruits for Transient Expression Analysis

For overexpression*, SlMADS-RIN* coding sequence was amplified from breaker stage cDNA and then cloned into the pENTR/D-TOPO donor vector (Invitrogen). It was further subcloned into the pK2GW7 gateway plant destination vector to generate a plasmid-designated as pK2GW7-SlMADS-RIN-OE, and then transformed into agrobacterium GV3101pmp90RK strains. Mature green fruits from wild-type and ethylene-deficient genotypes were chosen for agroinfiltration and subsequent expression analysis. Agrobacterium culture carrying pK2GW7-SlMADS-RIN-OE was grown overnight at 28°C in Luria-Bertani (Sigma) medium with selective antibiotics (gentamycin, rifampicin and kanamycin). Primary culture (500 μl) was then transferred to a 50-ml modified induction medium (2 mM MgSO_4_, 20 mM acetosyringone, 10 mM MES, pH 5.6) plus antibiotics, and grown overnight until optical density reached 1.0. The culture was centrifuged at 8,000 rpm for 5 min, resuspended in the infiltration medium (10 mM MgCl_2_, 10 mM MES, 200 mM acetosyringone, pH 5.6), and incubated at room temperature with gentle agitation (50 rpm) for a minimum of 2 h. Tomato agro-injection was done as described earlier (Orzaez et al., 2006). Briefly, 3–4 mature green tomato fruits from each genotype were infiltrated using a 2-ml syringe with needle (BD Biosciences). Needle was introduced to a depth of 3 to 4 mm into the fruit tissue through the stylar apex, and the infiltrated solution was gently injected into the fruit. The total volume of solution injected varied with the size of the mature green tomato fruit, from 600 μl to 1 ml. The completely infiltrated fruits were used for the experiments. Thereafter, fruits were harvested after 72–96 h of infiltration. RNA from agro-injected fruit samples was isolated and used to make cDNA as described previously (Shukla et al., 2017). cDNA was diluted 10-fold for qRT-PCR analysis. pK2GW7-*SlMADS-RIN* infiltrated fruits and control fruits infiltrated with infiltration medium were analyzed for the accumulation of *SlMADS-RIN*, *SlACS2* and both *sHSP* gene transcripts.

### Data Analysis and Statistics

Statistical analysis was carried out using Graph Pad (version Prism 8.0) and P values < 0.05 treated as statistically significant as described previously (Upadhyay and Mattoo, 2018). Significant differences between fruit ripening stages were calculated by separately comparing mature green/(-)5BR stage with breaker (BR), breaker+3 (BR+3), and breaker+8 (BR+8) stages for each gene. Similarly, significant differences between wild-type and ripening stages of mutants were calculated by comparing BR, BR+3 and BR+8 stages of *AC/AC* with respective stages of *rin/rin*, *nor/nor*, and *Nr/Nr*. Significant changes for *SlHSP17.7* transcripts in the *in vitro* ethylene suppression experiment were calculated by comparing air and ethylene treated samples at 12 and 24 h.

## Results

### Identification and Phylogenetic Analysis of Tomato *SlHSP17.7A, B* Class I HSP Genes

**Figure 1:**
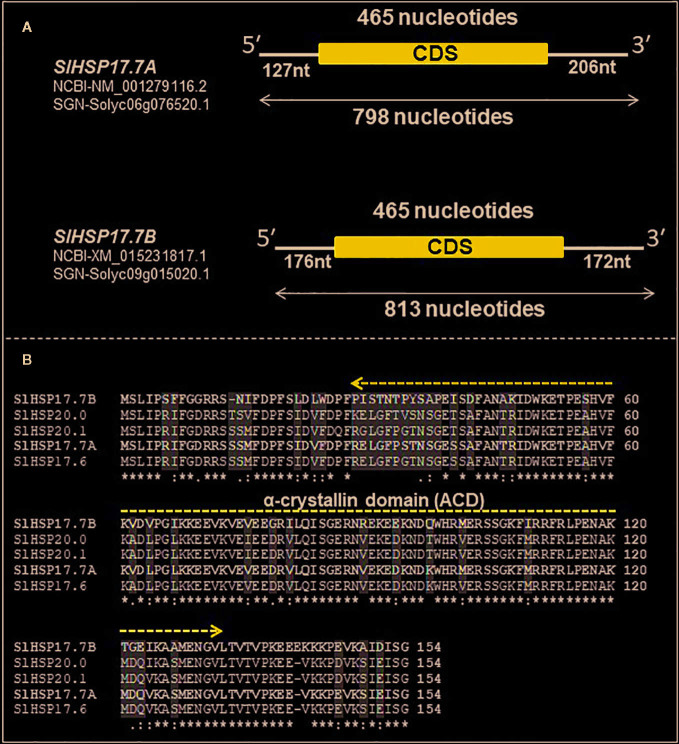
Organization, alignment and phylogeny of *SlHSP17.7A* and *SlHSP17.7B* genes. **(A)** Schematic representation of heat shock protein (*HSP*) genes with coding DNA sequence (CDS) and untranslated regions (UTRs). *SlHSP17.7A* gene (NM_001279116.2/Solyc06g076520.1) is made of 798 nucleotides with a CDS of 465 nucleotides, and 127 nucleotides of 5′ UTR, and 206 nucleotides of 3′ UTR. The *SlHSP17.7* CDS encodes a functional protein of 154 amino acids. Similarly, *SlHSP17.7B* gene (XM_015231817.1/Solyc09g015020.1) is made of 813 nucleotides with CDS of 465 nucleotides, and 176 nucleotides of 5′ UTR and 172 nucleotides of 3′ UTR. The *SlHSP17.7B* CDS encodes a functional protein of 154 amino acids. **(B)** Alignment of *SlHSP17.7A* and *SlHSP17.7B* to their closest homologs *SlHSP17.6*, *SlHSP20.0* and *SlHSP20.1*—different key conserved amino acid positions are shown. The characteristic conserved α-crystallin domain (ACD) is highlighted by dark blue line. Conserved residues are denoted by asterisks.

Bioinformatics analysis carried out as described in the Materials and Methods section revealed two novel class I intron-less sHSP genes which are organized similarly to a cluster of three sHSP genes described previously (Goyal et al., 2012). The two novel proteins were named SlHSP17.7A (Solyc06g076520.1.1) and SlHSP17.7B (Solyc09g015020.1.1), respectively. While this work was in progress, two genome-wide studies identified HSP20-related gene family in tomato (Paul et al., 2016; Yu et al., 2016). These studies indicated that *SlHSP17.7A* was housed on chromosome 6, and its close relative was identified as *SlHSP17.7B* housed on chromosome 9 of tomato. *SlHSP17.7A* gene (NM_001279116.2) constitutes 798 nucleotides with a coding DNA sequence (CDS) of 495 nucleotides with 127 nucleotides of 5′ UTR and 206 nucleotides of 3′ UTR. It encodes a functional protein of 154 amino acids, with a predicted molecular weight of 17,735.12 Daltons and PI 5.84 (**Figure 1A**). Like the *SlHSP17.7A* gene, the *SlHSP17.7B* gene (XM_015231817.1) constitutes 813 nucleotides with a coding DNA sequence (CDS) of 495 nucleotides along with 176 nucleotides of 5′ UTR and 172 nucleotides of 3′ UTR. The *SlHSP17.7B* CDS encodes a functional protein of 154 amino acids, with a predicted molecular weight of 17,662.01 Daltons and PI 5.84 (**Figure 1A**). The gene bank sequences for *SlHSP17.7A* (NM_001279116.2) and *SlHSP17.7B* (XM_015231817.1) were annotated with both untranslated regions (UTRs) while SGN sequence for *SlHSP17.7A* (Solyc06g076520.1) has yet to be annotated for positioning the 5′ and 3′ UTRs. Further, neither *SlHSP17.7A* (NM_001279116.2), (submitted in gene bank, Fray et al., 1990), nor *SlHSP17.7 B* (XM_015231817.1) have been so far characterized.

**Figure 2:**
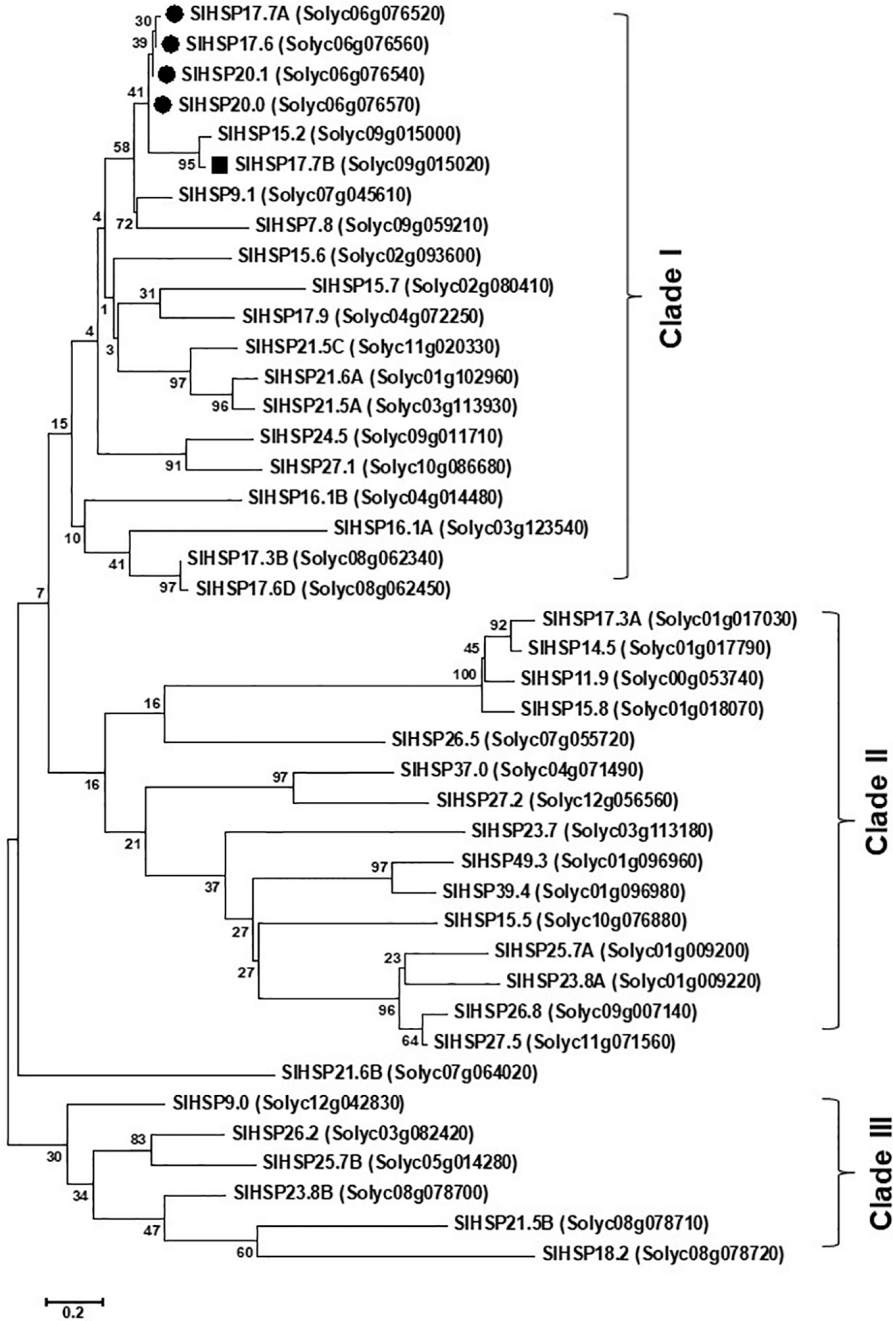
Phylogeny relatedness of SlHSP17.7A and SlHSP17.7B with other known proteins. The phylogenetic tree was constructed by maximum likelihood method with 1,000 boot strap values. Poisson model with complete deletion was employed during construction of the tree. The tree is drawn to scale, with branch lengths measured in the number of substitutions per site (Kumar et al., 2016). Forty two small heat shock proteins (HSPs) from tomato fell into two major groups, I and II. Group I was further segregated into “A” and “B” subgroups while group II was segregated into “C” and “D” subclades. *SlHSP21.5C* did not fall in any of the major groups. Subclade ‘A’ of group I consisted of small class I heat shock proteins namely, *SlHSP17.6*, *20.0*, and *20.1* (Goyal et al., 2012; Shukla et al., 2017) and the newly identified *SlHSP17.7A* and *SlHSP17.7B*.

Alignment of protein sequences revealed that both the SlHSP17.7A and SlHAP17.7B proteins differ from the other three close homologs, SlHSP17.6, SlHSP20.0 and SlHSP20.1, at 14 amino acid positions (namely, 14, 16, 27, 29, 34, 35, 36, 41, 62, 76, 98, 102, 124, and 146) (N→C) (**Figure 1B**). To decode evolutionary relationship among tomato sHSPs, a total of 42 protein sequences were extracted from SGN database and were exclusively annotated as small HSP proteins in tomato genome (Goyal et al., 2012; Yu et al., 2016) to construct a phylogenetic tree using MEGA7 program (**Figure 2**). The sequences separated into 3 major groups/clades. Both the SlHSP17.7A and SlHAP17.7B proteins constitute to clade I along with other members of this cluster, viz., SlHSP17.6, SlHSP20.0 and SlHSP21.0 (Shukla et al., 2017).

### Fruit Ripening-Specific Expression of *SlHSP17.7A* and *SlHSP17.7B* Is Synergistic With Upregulation of *SlMADS-RIN* and *SlACS2* Gene Transcripts

**Figure 3:**
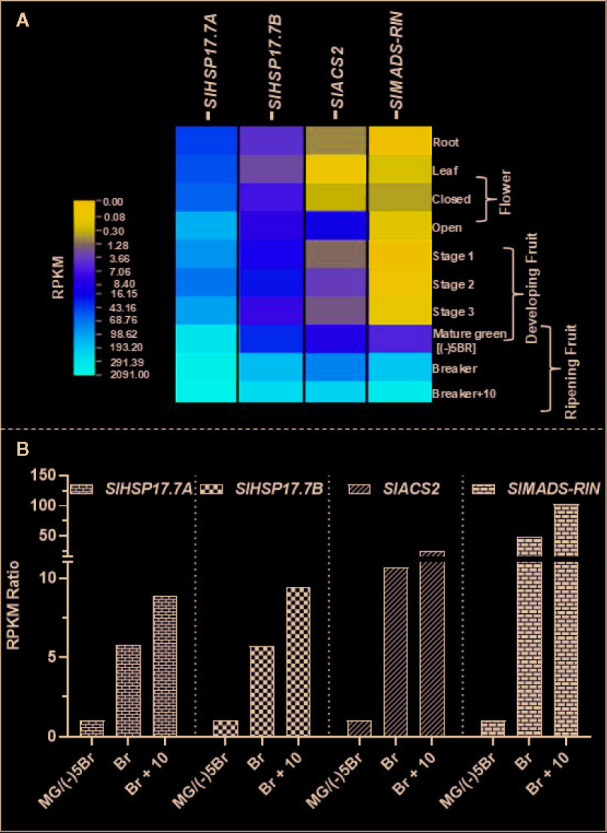
Transcriptome analysis of *SlHSP17.7* transcripts in wild-type *S. lycopersicum* cv. Heinz tomato during fruit ripening. **(A)** RPKM values for the *SlHSP17.7A*, *SlHSP17.7B SlACT2*, and *SlRIN* genes in tomato during plant growth, devepolment and fruit ripening [root, leaf, bud, flower, 3 fruit developmental stages, mature green (MG), breaker (BR), and red ripe (BR+10)] were derived from RNA-seq data from the SGN database (*S. lycopersicum* cv. Heinz) as described in the materials and methods section. **(B)** Mature green stage/(-)5BR was used as calibrator to reveal ripening-specific changes in gene expression. Fold change for each gene was calculated by dividing the RPKM values of (-)5BR stage to BR and BR +8 stages. Ripening-induced gene expression seen for *SlHSP17.7A* and *SlHSP17.7B* was corroborated with the expression of climacteric ethylene regulator *SlACS2* and *SlMADS-RIN*.

**Figure 4:**
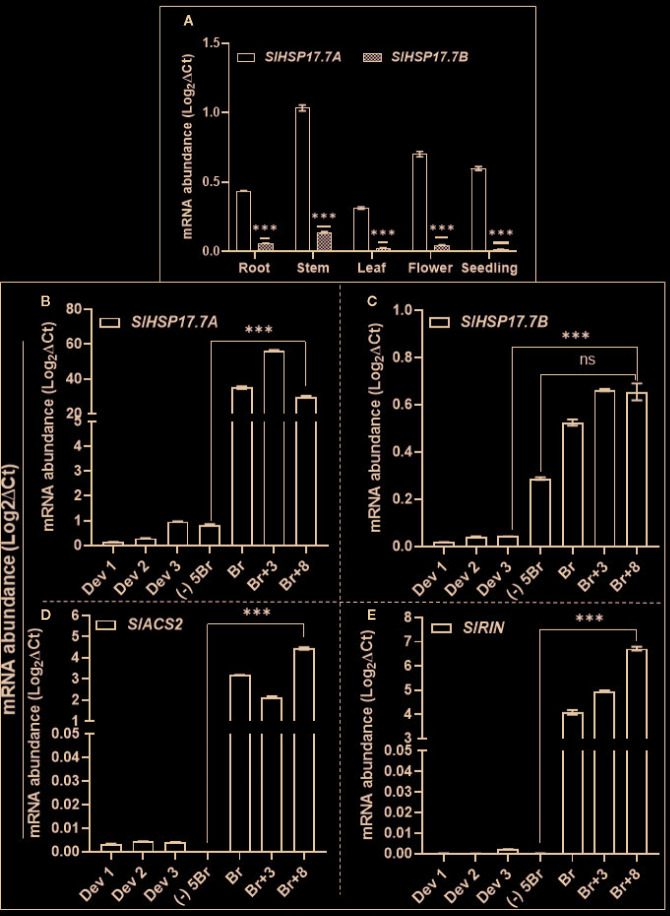
Quantitative real-time PCR (qRT-PCR) of *SlHSP17.7A, SlHSP17.7B, SlACS2 and SlMADS-RIN* genes in different tissues of wild-type *S. lycopersicum* cv. Ailsa Craig. RNA was isolated from root, stem, leaf, flower, seedling tissue and from fruit developmental and ripening stages. Leaf and stem samples were from 1-month old tomato plants; flowers were sampled from 3-month old plant; young seedlings were harvested from 8- to 10-day-old germinated seeds while for fruit harvesting stages Ailsa Craig plants were tagged at anthesis and fruits harvested at 8DPA (DPA = days post anthesis) (Dev 1), 15DPA (Dev 2), 22DPA (Dev 3) and ripening stages [(-)5BR, BR, BR+3, BR+8]. **(A)** Expression of *SlHSP17.7A* and *SlHSP17.7B* genes in vegetative tissue; **(B)** expression of *SlHSP17.7A* and **(C)** *SlHSP17.7B* during fruit development and ripening; **(D)** expression of *SlACS2* and **(E)** *SlMADS-RIN* during fruit development and ripening. *SlTIP41* and *SlUBI3* genes were used to normalize the expression of the target genes (Expósito-Rodríguez et al., 2008; Mascia et al., 2010). Error bars indicate standard deviation from a minimum of three biological replicates. Asterisks indicate statistically significant differences, *P < 0.05, **P < 0.01, ***P < 0.001 and ****P < 0.0001 (see Materials and Methods). ns, not significant.

We extracted available transcriptome data (*S. lycopersicum* cv. Heinz) from International Tomato Genome Sequencing Consortium (SGN; solgenomics.net) database (version ITAG 2.4) in order to determine the relative transcript abundances for *SlHSP17.7A*, *SlHSP17.7B, SlMADS-RIN* and *SlACS2* during plant development and tomato fruit ripening (**Figure 3A**). Gene transcripts of all these four are upregulated during ripening with the abundance of each gene transcripts increasing from MG/(-)5BR to BR stage [5.8-fold for *SlHSP17.7A,* 5.7-fold for *SlHSP17.7B,* 10.7-fold for *SlACS2*, and 49.3-fold for *SlMADS-RIN*] and yet more abundant at the BR+10 stage [8.9-fold for *SlHSP17.7A,* 9.4-fold for *SlHSP17.7B,* 24.1-fold for *SlACS2*, and 102.9-fold for *SlMADS-RIN*] (**Figure 3B**). Their RPKM counts followed an increasing trend with *SlHSP17.7A* > *SlHSP17.7B* > *SlMADS-*RIN > *SlACS2*, which indicates that the relative expression of *SlHSP17.7A* was highest among the four genes tested. In terms of fold change from mature green to ripening stages, *SlMADS-RIN* transcripts were highly expressed, followed by *SlACS2* > *SlHSP17.7B* > *SlHSP17.7A*. The transcriptome data for *SlHSP17.7* genes expression was validated by qRT-PCR analysis of RNA from vegetative tissues (root, stem, leaf, flower and seedling) (**Figure 4A**) and fruit developmental/ripening stages of Ailsa Craig (**Figures 4B, C**). Overall, the expression of *SlHSP17.7A* and *B* genes was found low in the vegetative tissues and during fruit development as compared to that during fruit ripening progression. Both sHSPs were similarly expressed in the stem while in leaf, flower and seedling tissues expression of *SlHSP17.7B* was significantly lower (P < 0.001) compared to that of *SlHSP17.7A*(**Figure 4A**).

During fruit ripening both the sHSP genes were upregulated as is known for the ripening regulators *SlACS2* and *SlMADS-RIN*. In Ailsa Craig, *SlHSP17.7A* expression was 40.63-fold higher at BR stage, increasing significantly (P < 0.001) to 61.56-fold at BR+3 stage, and decreased thereafter to 35-fold as compared to MG (-)5BR fruit (**Figure 4B**). In comparison, *SlHSP17.7B* expression was 1.8-fold higher at BR stage (P > 0.01), increasing to 2.3-fold (P < .001) at BR+3 and 2.27-fold (P < .01) at BR+8 stage compared to the MG/(-)5BR fruit (**Figure 4C**). Notably, *SlACS2* expression in Ailsa Craig fruit increased from 18 thousand-fold at the BR stage to 25 thousand-fold (P <0.001) at the BR+8 stage as compared to the MG/(-)5BR fruit (**Figure 4D**). Similarly, *SlMADS-RIN* expression increased from 26 thousand-fold (P <0.001) at the BR stage to 43 thousand-fold (P <0.001) at the BR+8 stage as compared to the MG/(-)5BR fruit (**Figure 4E**). Similar high expression dynamics of *SlACS2* and *SlMADS-RIN* genes are known (Martel et al., 2011).

### Differential Accumulation of *SlHSP17.7A* and *SlHSP17.7B* Gene Transcripts in Alisa Craig Versus Ohio 8245 During Ripening

**Figure 5:**
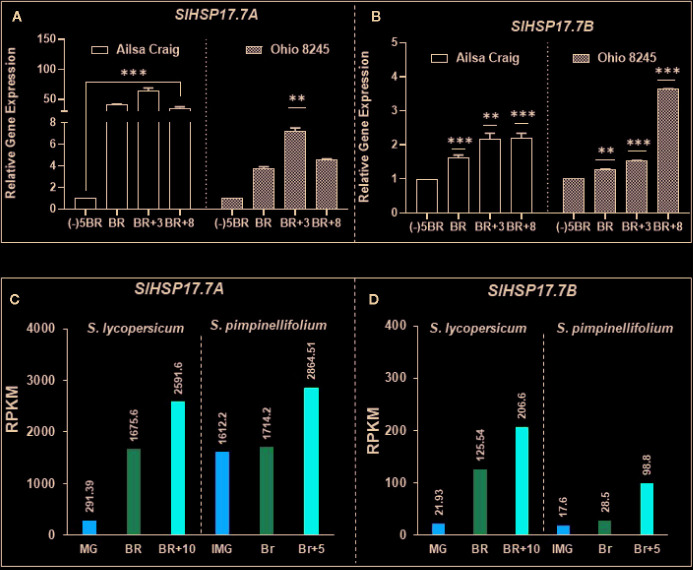
Differential expression of *SlHSP17.7A and SlHSP17.7B* genes in two modern tomato varieties as well as in two different genotypes. Tomato (*S. lycopersicum*) var. Ailsa Craig and Ohio 8245 fruits from various ripening stages were harvested as described in the Materials and Methods section. Both **(A)** *SlHSP17.7A* and **(B)** *SlHSP17.7B* genes showed differential expression during ripening. Variety-based abundance was also determined. Calibrator used for quantitative real-time PCR (qRT-PCR) calculations was (-)5BR stage. *SlTIP41* and *SlUBI3* genes were used to normalize the expression of target genes as described in the legend to **Figure 3**. Three biological replicates were used from each variety. A minimum of three fruits (n=3) were used for RNA isolation for each biological replicate. Transcriptome data for *Solanum lycopersicum* Heinz and *Solanum pimpinellifolium* fruit ripening stages for *SlHSP17.7A* and *B*, obtained from tomato expression database, show fold difference variation among genotypes with a functionally conserved ripening-induced nature of both HSPs **(C**, **D)**.

Ripening-regulated expression of *SlHSP17.7A* and *SlHSP17.7B* genes in Ailsa Craig was notably higher than in the Ohio 8245 variety (**Figures 5A, B**). Expression of *SlHSP17.7A* in Ailsa Craig fruit was 40.97-fold higher at BR stage, further increased by 64.87-fold at BR+3, and decreased thereafter to 35.65-fold at BR+8 compared to that at MG/(-)5BR. In comparison, *SlHSP17.7A* transcript expression in Ohio8245 genotype was notably much lower with a 3.7-fold increase at the BR stage, increasing further to 7.3-fold at BR+3 and 4.5-fold at BR+8 relative to that at MG/(-)5BR stage (**Figure 5A**). The expression of *sHSP17.7B* in Ailsa Craig and Ohio8245 fruits during ripening was not as dramatically different as that found for *SlHSP17.7A* gene above. In Ailsa Craig fruit stages, expression of *sHSP17.7B* was 1.63-fold at BR, 2.18-fold at BR+3 and 2.20-fold at BR+8 stages as compared to that in the MG/(-)5BR fruit. Likewise, in Ohio 8245 fruit stages, a moderate sequential increase in *SlHSP17.7* transcript expression was 1.26-fold at BR, 1.5-fold at BR+3 and 3.6-fold at BR+8 stages as compared to MG/(-)5BR stage (**Figure 5B**). Thus, the expression levels of these sHSP genes vary to different degrees in the two tomato varieties; however, in both varieties, the two genes are upregulated during fruit ripening.

Transcriptome data for *Solanum lycopersicum* Heinz and *Solanum pimpinellifolium* from tomato expression database also indicated that HSP expression greatly depends on the tomato genotype. However, a consistent ripening-induced expression of *SlHSP17.7A, B* genes is observed irrespective of genotypes (**Figures 5C, D**). This also reflects a functional conservation of these genes during evolution irrespective of gain in fruit size between S. *lycopersicum* vs. S. *pimpinellifolium.*

### Ethylene Inhibits *SlHSP17.7A, B* Expression in a Time-Dependent Manner in Fruit and Leaf Tissues

**Figure 6:**
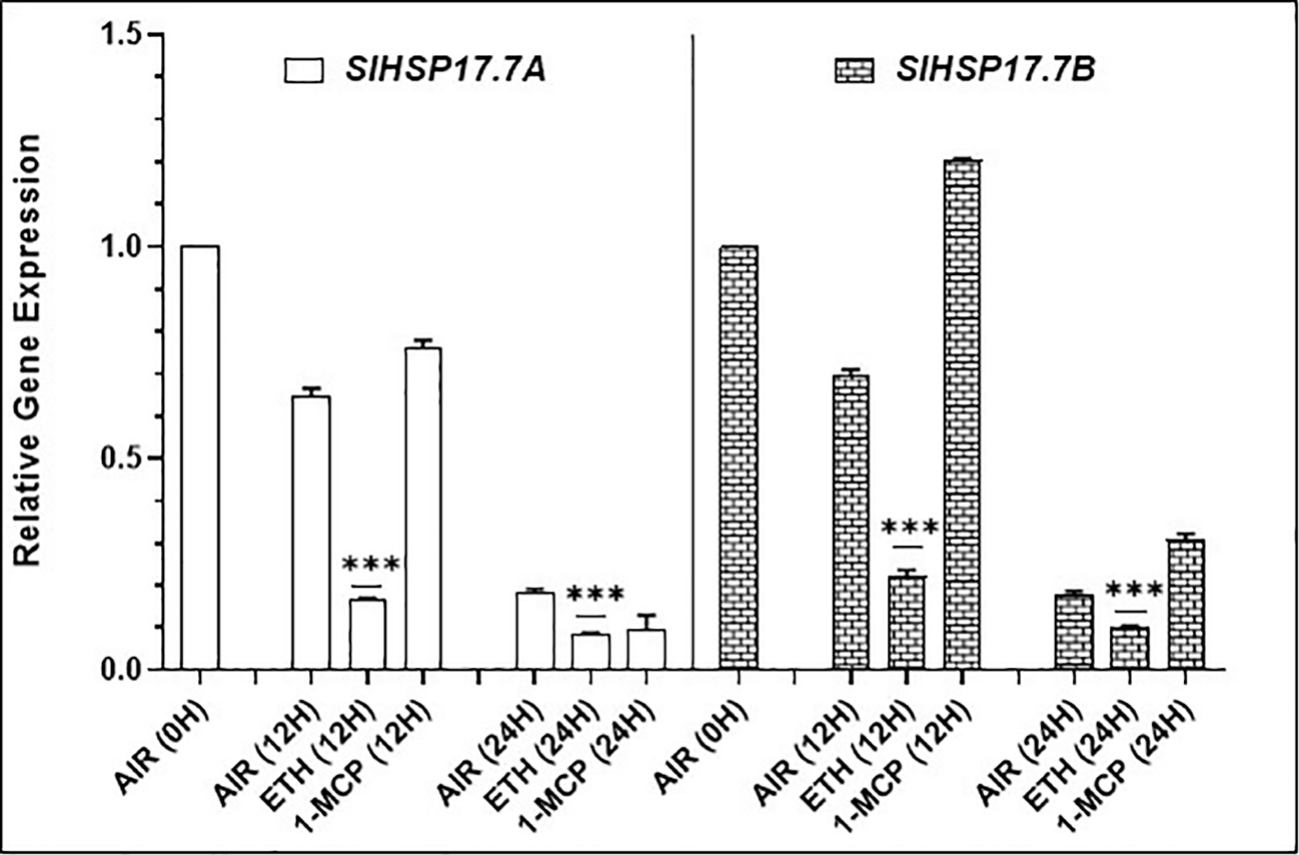
Effects of ethylene (ETH) and 1-methylcyclopropene (1-MCP) treatments on the expression of *SlHSP17.7A* and *SlHSP17.7B* genes. Wild-type (WT) (Ohio8245) fruits at (-)5BR/mature green stage were treated with either 25 ppm ethylene, 2 ppm 1-MCP, or left in air. Samples were then harvested at 0, 12 and 24 h, total RNA was isolated and expression of *SlHSP17.7A* and *SlHSP17.7B* genes was quantified. Error bars indicate standard deviation from a minimum of three replicates. *SlTIP41* and *SlUBI3* genes were used to normalize the expression of the target genes as described in the legend to **Figure 3**. Statistical differences [*P ≤ 0.05, **P ≤ 0.01, ***P ≤ 0.001, and ****P ≤ 0.0001] are indicated.

To discern if ethylene treatment of fruit modulates the expression of these two sHSP genes, we held the Ohio8245 MG/(-)5BR fruits separately in either air (control), ethylene or in the presence of 1-MCP an inhibitor of ethylene signaling (**Figure 6**). Fruits held in ethylene atmosphere for 12 and 24 h were found to be suppressed for the expression of both *SlHSP17.7A* and *SlHSP17.7B* genes (P < 0.001). However, it took 24 h before the fruits held in 1-MCP were found to be suppressed in *SlHSP17.7A* expression (**Figure 6**). Notably, 1-MCP-treated tomato fruit had higher expression levels of *SlHSP17.7B* gene at 12 and 24 h as compared to air control. We also determined if ethylene-mediated suppression of these two sHSPs occurs also in leaves. Both the gene transcripts were found suppressed for a longer duration in tomato leaves incubated in the ethylene atmosphere (**Supplementary Figure 1**). This demonstrates that ethylene mediated transient suppression of both these sHSP transcripts occurs in both fruit and leaf tissues.

### Ripening Mutants Are Compromised in *SlHSP17.7A* Expression but *SlHSP17.7B* Expression Is Differentially Elevated During Ripening as Compared to the Wild Type

**Figure 7:**
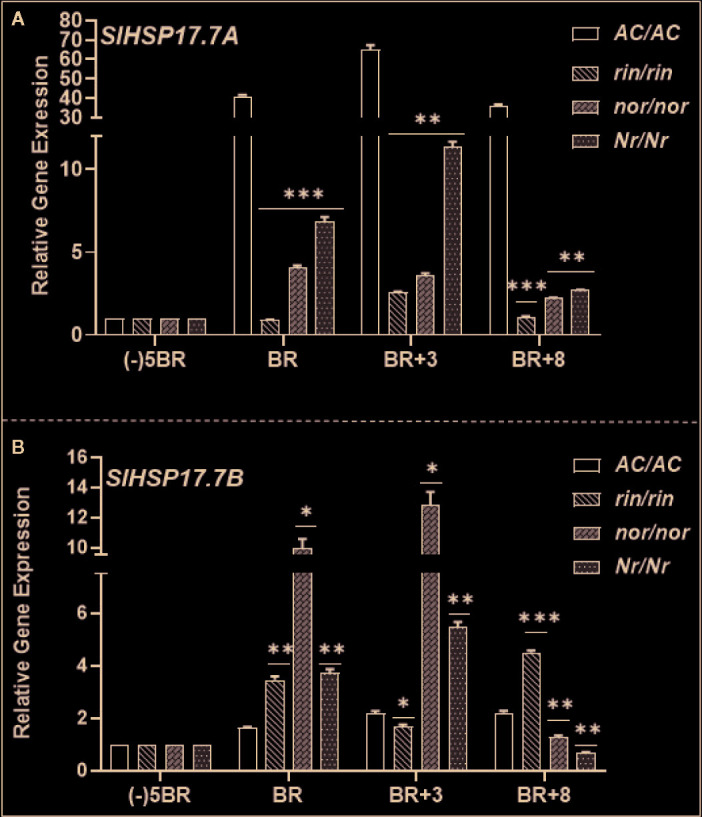
Expression patterns of **(A)** *SlHSP17.7A* and **(B)** *SlHSP17.7B* genes in nonripening tomato mutants. Fruits from wild-type tomato (*S. lycopersicum*) var. Ailsa Craig (*AC/AC*) and its ripening mutants (*rin/rin*, *nor/nor*, and *Nr/Nr*) were harvested as described in Materials and Methods section. quantitative real-time PCR (qRT-PCR) analysis, calibrator used for qRT-PCR calculations, and normalization of expression were the same as described in the legends to **Figure 5**. Statistical differences [*P ±0.05, **P ≤ 0.01, ***P ≤ 0.001, and ****P ≤ 0.0001] are indicated.

Expression of *SlHSP17.7A* and *SlHSP17.7B* genes was also quantified in the near isogenic nonripening mutant lines - *ripening-inhibitor (rin/rin)*, *nonripening (nor/nor)*, and *Never-ripe (Nr/Nr)* at similar age/ripening as the WT-Ailsa Craig (**Figure 7**). *SlHSP17.7A* transcript abundance was significantly impacted in the *rin/rin* and *nor/nor* mutants during the progression of ripening as compared to the WT-Ailsa Craig (**Figure 7A**). Similar was the case with *Nr/Nr* mutant fruit except that *SlHSP17.7A* transcript abundance was significantly higher at BR and BR+3 than in *nor/nor* and *rin/rin* mutants vis a vis WT-Ailsa Craig fruit (**Figure 7A**). An opposite trend in the expression of *SlHSP17.7B* gene in the three mutant lines was apparent as compared to WT. *SlHSP17.7B* gene expression remained high in mutants as ripening progressed (in *rin/rin*: 2.5-fold higher at BR and Br+8; in *nor/nor*: 5 to 6-fold higher at BR and BR+3 but lower at BR+8 stage; in *Nr/Nr:* 2-fold higher at BR and BR+3 stages but lower at BR+8) in comparison to the same ripening period of the WT-Ailsa Craig fruits (**Figure 7B**).

Since these tomato mutants are nonripening in nature and deficient in ethylene biosynthesis and perception, any deviation in their expression from wild type would be considered as ripening-specific and ethylene dependent. These results indicate that while *SlHSP17.7A* has ripening-specific expression, expression of *SlHSP17.7B* is not limited or regulated by ripening.

### Differential Regulation of *SlHSP17.7* and *SlMADS-RIN* Expression in ACS2 Gene-Silenced Transgenic Tomato

**Figure 8:**
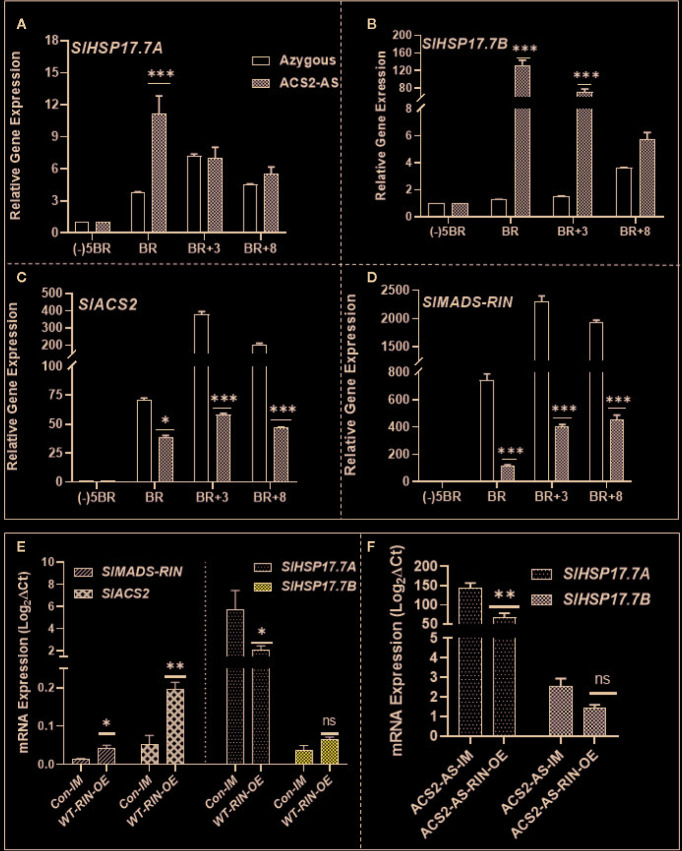
Expression dynamics of *SlHSP17.7A*, *SlHSP17.7B*, and *SlMADS-RIN* during fruit ripening of ACC synthase 2 (*SlACS2*)-silenced tomato transgenic line (ACS2-AS) and the azygous/wild-type control lines (WT-Ohio8245) and RIN agroinjection. Fruit at different ripening stages were harvested from ACS2-AS and azygous control plants. RNA was isolated and expression levels of **(A)** SlHSP17.7A; **(B)** SlHSP17.7B; **(C)** SlACS2; **(D)** SlRIN were determined by qRT-PCR analysis. **(E)** Wild-type mature green tomato fruits were infiltrated with agrobacterium culture carrying RIN overexpression construct as described in the Materials and Methods Section. Fruits injected with only the infiltration medium were taken as control. RNA was isolated from infiltrated fruits and cDNA was prepared. Expression studies were done for *SlACS2, SlMADS-RIN*, *SlHSP17.7A*, *and SlHSP17.7B.* **(F)** Similar methodology as in (A) was repeated for ACS2-AS tomato and expression of *SlHSP17.7A* and *SlHSP17.7B* was determined. Error bars indicate standard deviation from a minimum of three replicates for each data point and statistical differences [*P ≤ 0.05, **P ≤ 0.01, ***P ≤ 0.001 and ****P ≤ 0.0001] are indicated.

To differentiate ethylene regulation of the two sHSP genes and of *SlMADS-RIN,* we employed our previously characterized transgenic tomato line ACS2-AS silenced for ACS2 gene which produces 50% less ethylene than the wild type (Sobolev et al., 2014). Expression of both, *SlHSP17.7*A and *SlHSP17.7B* genes, was upregulated only at the breaker stage compared to the azygous control line; however, this upregulation was many-fold evident in the *SlHSP17.7B* gene right from breaker stage to BR+8 stage (**Figures 8A, B**). Interestingly, the ACS2-AS ethylene-deficient line was also found to be compromised in the expression of the ripening-regulator *SlMADS-RIN* gene (**Figures 8C, D**). These data invoke ethylene as a negative regulator of the two sHSPs and, likely, the *SlMADS-RIN* gene.

### *In Silico* Analysis of the *SlHSP17.7A, B* Gene Promoters and Identification of Various *cis*-Elements

We also analyzed the 2 Kb upstream 5′ putative promoter regions of the two tomato *SlHSPs* genes for the presence of ethylene-related cis-elements ERE (ethylene response elements) and RIN binding ‘CArG’ box *cis*-elements using PLACE (Higo et al., 1999) and Plant Care (Lescot et al., 2002) databases. Several binding sites responsive to hormonal and environmental signals were found in the promoter of both the HSP genes (**Supplementary Tables 3** and **4**). Notably, two ‘CArG’ binding *cis*-elements in the promoters of each gene were found. The *SlHSP17.7A* promoter was decorated with one “atypical” [C(A/T)_8_G] motif type at -29 and other “possible” [C(C/T)(A/T)_6_(A/G)G] motif type at -1841 position (**Supplementary Table 3**). Similarly, *SlHSP17.7B* gene promoter was found decorated with both “atypical” [C(A/T)_8_G] motif types at -633 and at -1067 positions (**Supplementary Table 4**). Previously, CArG motifs have been shown to be target binding sites for *SlMADS-RIN* transcription factor to regulate fruit ripening genes (Martel et al., 2011; Fujisawa et al., 2013; Shukla et al., 2017). The presence of CArG motifs in *SlHSP17.7A* and *SlHSP17.7B* gene promoters suggests that *SlMADS-RIN* transcription factor may bind these *cis*-elements and regulate expression of HSPs during fruit ripening.

### Transient Overexpression of *SlMADS-RIN* Transcription Factor Reveals Differential Expression of *SlHSP17.7A* and *SlHSP17.7B* Genes in WT Versus ACS2-AS Tomato Line

Tomato fruits were infiltrated with pK2GW7-*SlMADS-RIN-*OE construct and, after 72–96 h of incubation period, infiltrated fruits were analyzed for the abundance of *SlMADS-RIN* and *SlACS2* transcripts as described in the methods section. Transcripts of both *SlMADS-RIN* and *SlACS2* were found upregulated, which indicated successful activation of *SlACS2* gene (3-fold; P < 0.01) by *SlMADS-RIN* transcription factor (**Figure 8E**). To check if *SlMADS-RIN* mediates suppression or activation of the two sHSPs, this cDNA was analyzed for the status of both the HSP genes. Interestingly, *SlHSP17.7A* expression was significantly suppressed (P=0.0023), and the *SlHSP17.7B* transcripts were activated but not to an extent to be statistically significant.

Finally, we also agro-injected the ACS2-AS transgenic tomato line (with low ethylene background) with the pK2GW7-*SlMADS-RIN-*OE construct. This experiment revealed a lower transcript abundance of *SlHSP17.7A* (P=0.0069); however, the *SlHSP17.7B* transcripts also were downregulated but not to a level to be statistically significant (**Figure 8F**). These results demonstrate that *SlHSP17.7A* transcripts are suppressed by *SlMADS-RIN* in different genetic backgrounds. These results are in tune with the previous findings where *SlMADS-RIN* was shown to regulate some genes in both ethylene-dependent and independent manner, reminiscent of the existence of negative as well as positive regulation of tomato genes during the transition of mature green fruit into ripening progression (Fujisawa et al., 2013).

## Discussion

Two novel class I small HSP genes (*SlHSP17.7A* and *SlHSP17.7B*) were identified in tomato and their transcriptional regulation was found to be mediated by the plant hormone ethylene. Both the sHSP genes share sequence similarities with other tomato class I small HSPs sequences (Goyal et al., 2012; Paul et al., 2016; Yu et al., 2016; Shukla et al., 2017). However, they are localized in different chromosomes in tomato, *SlHSP17.7A* gene is localized to chr. 6 in a tandem repeated manner with a previously characterized cluster of three small HSP genes *SlHSP17.6*, *SlHSP20.0* and *SlHSP20.1* (Goyal et al., 2012; Shukla et al., 2017). However, the *SlHSP17.7B* gene is resident on chr. 9 but shares a close homology to *SlHSP17.7A*. Both the s*HSP* genes are ripening-specific with minimal expression in the vegetative tissues; moreover, the *SlHSP17.7B* transcripts were observed at the developmental stage 3 of tomato fruit, a stage earlier than the mature green fruit. These two sHSPs can be further differentiated by their expression patterns in the ethylene-deficient tomato mutants (*rin/rin*, *nor/nor*, and *Nr/Nr*), with *SlHSP17.7A* gene expression characteristically impaired in these mutants while *SlHSP17.7B* gene is abundantly expressed in them.

A mutation in the ripening-regulator MADS-RIN protein prevents the tomato ripening mutants, particularly *rin*, from ripening (Vrebalov, 2002) since MADS-RIN protein regulates the expression of ethylene biosynthesis genes *ACS2* and *ACS4* during fruit ripening (Cara and Giovannoni, 2008; Martel et al., 2011). This is also apparent from the data presented here showing that the *SlMADS-RIN* transcription is in congruence with the expression patterns of *SlACS2*. In this regard, our *in silico* analysis revealed that the promoters of both the *SlHSP17.7A* and *SlHSP17.7B* genes harbor at least two *MADS-RIN* binding *cis*-elements (**Supplementary Tables 3** and **4**), the CArG motif (Ito et al., 2008; Fujisawa et al., 2011; Zhong et al., 2013). Similar CArG motifs has been shown in promoters of other genes by previous workers. For example, CArG motif at position −1841 (CAAAAAAAAG) in gene promoter of *SlHSP17.7A* has been previously identified in Solyc04g082420 promoter as potential direct target of RIN transcription factor (Fujisawa et al., 2013). While, CArG motifs in *SlHSP17.7B* promoter at position −633 (CTTAAATATG) and −1067 (CATTAATTTG) has been previously shown as direct target of RIN in Solyc01g104050 and Solyc09g065030 gene promoters, respectively (Fujisawa et al., 2013). This further strengthen a possibility of RIN transcription factor binding with CArG motifs present in HSP gene promoters. Thus, we envisioned an interaction of RIN with promoter elements of the two sHSPs and tested this hypothesis *via* transient expression of RIN in WT and ethylene-deficient tomato line. Significant suppression was apparent for *SlHSP17.7A* gene but not the *SlHSP17.7B.* Thus, *SlMADS-RIN* may be a negative regulator of *SlHSP17.7A* gene.

Together, these data reinforce the view that some small HSPs regulate fruit ripening in tomato. Thus, ethylene and transcription of class-1 sHSP genes seem interlinked in tomato, particularly at the initial phase of fruit ripening. These findings also corroborate the suggestion put forward earlier that observation of ripening induced expression of some HSP ESTs (Fei et al., 2004) and another class-I s*HSP21* gene may be crucial for progression of fruit ripening (Neta-Sharir et al., 2005). This indicates that additional factor(s) are involved in inducing these sHSP genes during fruit ripening. Our data showing that ethylene-treated tomato leaf is suppressed in the expression of *SlHSP17.7* gene suggests that this regulation occurs in both tissues, fruit and leaf. tissue independent.

We opine that ethylene suppression of *SlHSP* genes at the onset of fruit ripening when ethylene synthesis is initiated may be an indigenous regulation slated to enable fruit ripening to proceed uninterruptedly. Interestingly, transient suppression of both *SlHSP17.7A* and *SlHSP17.7B* genes by exogenous ethylene was more robust in the ethylene-deficient ACS2-AS genotype. Since ACS2-AS transgenic line produces only 50% of ethylene relative to its control suggests that a certain threshold of ethylene is necessary to achieve robust suppression of *SlHSP17.7* transcripts.

Our previous work on SlHSP17.6, 20.0, and 20.1 showed that transient suppression of these genes during fruit ripening transition is regulated by SlMADS-RIN-mediated and ethylene-dependent pathway (Shukla et al., 2017). Here, we demonstrated that *SlHSP17.7A* follows a similar kind of transcription regulation as the other members of this cluster (Goyal et al., 2012). However, *SlHSP17.8B* gene seems to be regulated by some other, as yet unknown transcriptional regulator different from SlMADS-RIN. However, in regard to regulation by ethylene both of these sHSPs are similarly suppressed by exogenous ethylene and their expression is highly upregulated at breaker stage in the ACS2-AS transgenic background tomato, a situation where SlMADS-RIN transcripts were found to be very low.

Notably, our results that demonstrated low SlMADS-RIN expression during fruit ripening in the ACS2-AS transgenic line deficient in ethylene by 50% suggest that (i) ethylene is required to induce SlMADS-RIN transcripts directly or/and (ii) an unknown ethylene-dependent regulator is necessary to induce SlMADS-RIN during fruit ripening transition. These results suggest the need for a reevaluation of the role of SlMADS-RIN as the master regulator of fruit ripening which is also recently studied by different groups (Ito et al., 2017; Wang et al., 2020). Although, this was not the primary focus of this study but our primary results indicate that still ethylene is upstream to RIN, albeit this hypothesis needs rigorous experimentation. Further functional genomics studies are needed to characterize *in planta* the promoter of the two *SlHSP17.7* genes. Novel transgenic approaches can shed further light on specific role(s) of small HSPs in fruit physiology and ripening.

Differential gene expression in duplicated sHSP genes has been linked to homeostasis maintenance and their likely role(s) in responses to different stresses (Arce et al., 2018). Since HSP roles include protein folding, chaperone function and other, they are deemed essential for refolding proteins under abiotic stress situations (Murakami et al., 2004; Wang et al., 2004). Clearly, their committed role(s) in plant life and fruit ripening as presented here need to be followed further.

## Data Availability Statement

The raw data supporting the conclusions of this article will be made available by the authors, without undue reservation.

## Author Contributions

Conceived and designed the study: RU and AM. Performed the experiments: RU. Ethylene treatment of fruits and leaves: RU and MT. Analyzed the final data: RU and AM. Reagents availability: AM. Facilitated the research: AM. Wrote the paper: RU. Finalized the paper: AM.

## Funding

This research was supported by an USDA-ARS intramural Project No. 8042-21000-143-00D (PI. AM). Mention of trade names or commercial products in this publication is solely for the purpose of providing specific information and does not imply recommendation or endorsement by the U.S. Department of Agriculture.

## Conflict of Interest

The authors declare that the research was conducted in the absence of any commercial or financial relationships that could be construed as a potential conflict of interest.
