# Supplementary material for: Ethylene and RIPENING INHIBITOR Modulate Expression of SlHSP17.7A, B Class I Small Heat Shock Protein Genes During Tomato Fruit Ripening
Source: Front Plant Sci. 2020 Jun 30;11:975. doi: 10.3389/fpls.2020.00975 (PMC7344320; doi:10.3389/fpls.2020.00975)
Supplement: Supplementary file 1 [file DataSheet_1.docx]

**SUPPLEMENTARY FILES**

**Ethylene and *RIPENING INHIBITOR* modulate expression of *SlHSP17.7A, B* class I small heat shock protein genes during tomato fruit ripening**

**Rakesh K. Upadhyay^1^, Mark L. Tucker^2^ and Autar K. Mattoo^1^***

^1^Sustainable Agricultural Systems Laboratory, The Henry A. Wallace Beltsville Agricultural Research Center, United States Department of Agriculture-ARS, Beltsville, MD, United States

^2^Soybean Genomics and Improvement Laboratory, Agricultural Research Service, United States Department of Agriculture, Beltsville, MD, United States

^*^**Correspondence**: AKM ([autar.mattoo@usda.gov](mailto:autar.mattoo@usda.gov))

**Supplementary Tables and Figures**

**Supplementary Table 1:** Tomato heat shock protein sequences used for generating phylogenetic relationship among members

**Supplementary Table 2:** List of genes and their primer sequences used for quantitative real–time PCR (qRT-PCR) analysis

**Supplementary Table 3:** List of *cis-*elements present in *SlHSP17.7A* promoter analyzed by Plant Care and PLACE promoter databases (Higo et al., 1999; Lescot *et al*., 2002)

**Supplementary Table 4:** List of *cis-*elements present in *SlHSP17.7B* promoter analyzed by Plant Care and PLACE promoter databases (Higo et al., 1999; Lescot *et al*., 2002)

**Supplementary Figure 1.** Expression of *SlHSP17.7A* and *SlHSP17.7B* genes in response to ethylene (ETH) treatments to leaf tissues

**Supplementary Table 1: Tomato heat shock protein sequences used for generating phylogenetic relationship among members**

| >SlHSP11.9 (Solyc00g053740) MDRLDVQVVDVGPPADWVKINVRETNDSFEVYALVSGLSREEVRVQSDPAGRLVITGQPKQLDNFWGVTAFKKVVNLPARIDQLRTNTGVTLHGCLHVHVPFAQQNL* |
| --- |
| >SlHSP25.7A (Solyc01g009200) MNSKGATPTQVYEDFVPSSKKVHEENFDTIHINLHGFKKEQLKVVLTSTGTLKISGQRPIGPTKWQRFLNEFPVAENCDRSKISAKFENGILHVKQPKLIPKVEKEKELSATLAENTPAAKRQKTTLRDEFGKQDNADVDTPAKEEPKETSPKTSEQTEEKKLPSHDSSSSSSSHSSESDSESTDDDTDDEASENVSCLVANLKKPSKVIQMTLVSLLVLGISLYIANERSH* |
| > SlHSP23.8A (Solyc01g009220) MENGNKDNCSCCSNSRIHEDFMPTSELVHGKDYDTLLLNLPGFKKEEVKIQLCKRTGILKINGQRPVNKFLSFQKDIPVSKDCDKSKINARLVNGILYVRHPKLIISSQKYENDLPTSTNIEPKQDKKKTKLDEPSGQDNAGKPNNTSPKSNEQTQVVQRNGSMKDDATTTRTSSDIFAKLKVSRQVMNIALAALVVLGIGGYVMRFPKKAKE* |
| > SlHSP17.3A (Solyc01g017030) MVRFSPSKPCLLFLSPLSNIPSSLRCKGLCDCFHDLNHSRIFFRNVLLRLDVQVVDVGPPADWVKINVRATNDSFEVYALVPGLLRDELRVQSDPTGRLVITGHPHQLDNPWGVTSFKKVVILPSRIDHFRTNAILTFHGCLHVHVPFAQQNL* |
| > SlHSP14.5 (Solyc01g017790) MCKGSCDCVHELNQSRIFFRNVLLRLDVQVVDVGPPADWVKINVRATNDSFEVYALVPGLLRDELRVQSDPTGCLVITGQQHQLDNPWGVTSFKKVVTLPARIDQLRTNAVLTFHGCLHVHVPFAQQNL* |
| > SlHSP15.8 (Solyc01g018070) MVGSSLLPMTPFCLVLLPLIYSVCLGDILAAFAISLILLDVQVDDVGPSTDWVKINVRETNDFFEVFALVPWLSLDEVRVQSDPAGRLVITGQPKQLDNFWGVTSFKKVVTLPSRIDQLRTHADLTLHGCLHVHVPFAQQNL* |
| > SlHSP49.3 (Solyc01g096960) MAMRPRGGGVGPNRGPAARAVYEDFRPVHERHQDEEAEKLVIYLPGFMKENIRVSTEGKNTVRVRGERFVGGNKWHRFQEDFQAPDDCNMRGIHAKFENGILIITMPWKMPKQLADEHTKQSAPIIPHKDDNVPPRTTHPTVETPRKTTAQRADKDQDSTRNETSKTTYPTTEATPKKPTHLKPTAQLPKPQHDDKDKVSTRNETMGSAESSKTQNGDNVPPKTTYPTTQAAPRKPTPLKPTAQLPKPENTDNDQDSRVKEMKFFDDQLMSPESSKTQMGDEDIDFPALRATTLGKTSGEREKPKDEKKIFEGLVGSIEPKIQKSKEENLDQNRTQLIKSGKDVGKEESHDAGGKIVAEKIKDLREEFQEQVGQKGSKEKESTSNVAAGTSFYTGSISNLKKSIVELNEERQLLVNAGAAVMVIMALGTYVYYSIRSGRTE* |
| > SlHSP39.4 (Solyc01g096980) MATGPNRASPPVYEDFRPVSEWHQEAGAEILLIFLPGFMKESIRVSIEGKNTVRAGGELFIGSNTWCRFQEDFQAPDDCNIKRIDVKFEDGILTITVPRKRPRQLLDEQTEQFPHRIPHKDDNIPPTNNHSTDENPRKLPSLQPTTQHAHKDQDSTRNETLGSAESSNTQKGDKFPPRTTYPTTEAAPRKPTPVKPATQQPKHQHAHKDQDSTRNETSGSAESSNTQKGDKFPPRTTYPTTEAAPRKPTPVKPTAQQPKPQHAHKDQDSTRNETSRSAESSNTQKGDNVPSRTTYLNCQNLNILTKIKILQEMRHRGVLNHQILKRVIMFLPEQLMQLLKPPQENQPL* |
| > SlHSP21.6A (Solyc01g102960) MAKAKAHVMSFLVLATTILAILPSKTQALMPYTRPLFDLMFPQEDPFKILEQTPLTIPKGIDQTIALLARSDWKETSKEHIISLDIPGMKKEDIKIEVEENRVLRISGERKTEEENIESEKWHRVERTSGKFWRQFKLPRNVDLEHIKANLDNGVLKITVPKLAEEEKKQSKVISISEQVNGGDIKATM* |
| > SlHSP15.7 (Solyc02g080410) MEFSTFHPSTWNSFFTSPLLFPYQFIPENYVHWRETPESHIYSADLPGVKKEEIKVEVEDSSYLIIRTEAANETTEPIRSFMRKFRLPGMVDMDGISASYRDGVLTVTVPRTLVRRGFFIEPDDLTESIVNLGASAA* |
| > SlHSP15.6 (Solyc02g093600) MCSLLRSSDPIVGMMNMCPVLSTPIDWKETSQAHCFFVDLPGLSKEDVKVEVDNGRVVKISGKWKAEEEIGDENEKKNLWHRVERNRGDFCRKFRLPKNIMADRLEASMENGVLVLTVPKQQLKKPFSKVIEIEEK* |
| > SlHSP26.2 (Solyc03g082420) MAYTSLTSSPLVSNVSVGGTSKINNNKVSAPCSVFVPSMRRPTTRLVARATGDNKDTSVDVHHSSAQGGNNQGTAVERRPTRMALDVSPFGVLDPMSPMRTMRQMIDTMDRLFEDTMTFPGRNRASGTGEIRTPWDIHDDENEIKMRFDMPGLSKEDVKVSVENDMLVIKGEHKKEEDGRDKHSWGRNYSSYDTRLSLPDNVVKDKIKAELKNGVLFISIPKTEVEKKVIDVQIN* |
| > SlHSP23.7 (Solyc03g113180) MEHYGDHRRGTNLTTSVNPVYEDIEPSSGWIEDAENHYLLIDLPGFKREEVKLEVDTFDNIKVSGERKVGENKFIRFQKSTIAPEKSKSEDTSARIEDGILFVIIPKELPENNEREEAAIASSGHEENQQEEIESSKGHDTEEKENDKQGPNGDEEFHDAKMAKKWHEAYPVAAGKEILKKNKTIVITALLAFSFGVYVSQKWQSSKID* |
| > SlHSP21.5A (Solyc03g113930) MAKTRVSFMSFLVLAMVVVLFPSQIKALMPYTRPFWDIAFPPEDPFRILEQTPLTIPKGVESIALTRSDWKETATEHVITLDIPGMKKEDVKIEVEENRVLRVSGERKTEEEIEGEKWHRAERTCGKFWRQFRLPGNADLEHIKAHLENGVLKITVPKLAEEKKKQSKVISIAEAVGGEDIKANKAEM* |
| > SlHSP16.1A (Solyc03g123540) MSSVVDVVSQLLFPESIERLVSPSRSNESRGTIPVDILDTPKEYIFYMDVPGLSKSDIQVSVEDEKTLVIRSNGKRKREESEEEGCKYVRLERNPPLKLMRKFKLPDYCNVSAITAKCENGVLTVVVEKMPPPSKAKTVKVAVS* |
| > SlHSP16.1B (Solyc04g014480) MAIFGDPFRRFLLSPTIHRSFSGSPALLDWIESPNSHIFKINVPGYSKEDIKVQVEDGNVLVVKAEGHGGKKDEFHGKEKDIVWHVAERGGGRGGDFSREIELPEDVKVDQIKAQCENGVLTIVVPKDATPKTSKVRNINITSKL* |
| > SlHSP37.0 (Solyc04g071490) MEMEMGLKLTRVANESSSTEFQFAKDRAGPLFQSTETDTMFILTVHLKGYIQENIKVDINEEGTIIAIRGEKSVQETVMVGWKLIKKDVEVRKFSKAFKIPDGVILDEIKARFDDEISILTIKMPKKVKGILGIEFVEVKEHEELPIVANKISKKVTFKEDMDKPKAEENQGKPREVVQKHVVKDEMMPNEDSKHVVKDEIMPNEDSKHVVKDEMMSNEDSKHVIKDETMPNVDSNIQNNDTIDETREARGDFVGHDKPESSNSRDEHKDDQASVTSRKRED DNVPKKSSKICVPIVAGSALILSLFVFVIHLIRTKNQSVKRKG* |
| > SlHSP17.9 (Solyc04g072250) MASSIGPWLGGGGRSRDMDFVSPFSSDVLGLGFGGELGFGNGLGFRNDEISALAHASVDWRETDQAHVFLVDIPGVKKEDLKVQLEDNILEISGERVKEEEKGDDKWHRVERKRGSFCRKFRLPENANVEGISCGLENGVLTVNVPKKETQQVPKNVKAINIT* |
| > SlHSP25.7B (Solyc05g014280) MAHCLSRFPISQPILFSNNPKYSNSSLPISSRKYQGNYKKLKVMVVDERHNLDHLQRQNKTPQPRKRTPQMAPVGLWDRFPTARTVQQMMDTMDRVIEDPLAFNGGPSTDDIGYRRGRTPWEIKENEGEYKMRFDMPGMTKEDVKVWLEEKMLVVKGEKMVKNNEKEEEWSAKSYGKYNTRIALPENIDFEKIKAEVKDGVLYITIPKASSNPKVFDINVQ* |
| > SlHSP17.7A (Solyc06g076520) MSLIPRIFGDRRSSSMFDPFSIDVFDPFRELGFPSTNSGESSAFANTRIDWKETPEAHVFKVDLPGLKKEEVKVEVEEDRVLQISGERNVEKEDKNDKWHRMERSSGKFMRRFRLPENAKMDQVKASMENGVLTVTVPKEEVKKPEVKSIEISG* |
| > SlHSP20.1 (Solyc06g076540) MSLIPRIFGDRRSSSMFDPFSIDVFDQFRGLGFPGTNSGETSAFANTRIDWKETPEAHVFKADLPGLKKEEVKVEIEEDRVLQISGERNVEKEDKNDTWHRVERSSGKFMRRFRLPENAKMDQVKASMENGVLTVTVPKEEVKKPDVKSIEISG* |
| > SlHSP17.6 (Solyc06g076560) MSLIPRIFGDRRSSSMFDPFSIDVFDPFRELGFPGTNSGESSAFANTRIDWKETPEAHVFKADLPGLKKEEVKVEVEEDRVLQISGERNVEKEDKNDKWHRVERSSGKFMRRFRLPENAKMDQVKASMENGVLTVTVPKEEVKKPEVKSIEISG* |
| > SlHSP20.0 (Solyc06g076570) MSLIPRIFGDRRSTSVFDPFSIDVFDPFKELGFTVSNSGETSAFANTRIDWKETPEAHVFKADLPGLKKEEVKVEIEEDRVLQISGERNVEKEDKNDTWHRVERSSGKFMRRFRLPENAKMDQIKASMENGVLTVTVPKEEVKKPDVKSIEISG* |
| > SlHSP9.1 (Solyc07g045610) MENNWSTGETPAFANTRIDWKETPEAHLFKADLPGLKKEEVKVEIKEDRVLQISRERKVEKEDKNDTWHCVEQSNGNS* |
| > SlHSP26.5 (Solyc07g055720) MESQIVRRRVNMITAHLTAHDDISASATHLFPMSCSSSLNSAIPRRYDNRMNYARQSSSSQACFMRTSEQGSCTESTAAFKASDYAKKSSRAFEGPMFSRPANNCKHNGTVEEAPKFARPRFQLKERRNELESNGSEWSPKMDVAESGSMYVVSIELPGVNINDIKVEVSHKSLIVSGNRSTQCKVASYLNGLVSAYHKKEIVQGPYRVFWPLPSNANKNRVSAEFVDGLLQITIPKL* |
| > SlHSP21.6B (Solyc07g064020) MSKQLEVQFEESNPKKWCVPLKEDVFVKFMNKGNFITHKALCEGSFFSPLLFGKFFDPSDAFPLWEFDSDVLLSNAKSSNNNHYKVDWIQTETDYVLKTEIPGVGKGIICVSVEDGKVLEVSGQFRLKVETGTKDWRVGNWWEHGCVRRIELPENADWKKTEALMSNGDHKFLEVKIPKIPPNICDVP* |
| > SlHSP17.3B (Solyc08g062340) MDLRLMGIDNTPLFHTLQHMMEAAGEDSVNAPSKKYVRDAKAMAATPVDVKEYPDSYVFVVDMPGLKSGDIKVQVEEDNVLLISGERKREEEKEGVKFIRMERRVGKFMRKFSLPENANTDAISAVCQDGVLTVTVQKLPPPEPKKSKTIQVKVA* |
| > SlHSP17.6D (Solyc08g062450) MDLRLLGIDNTPLFHTLHHMMEAAGEDSDKSVNAPSRNYVRDAKAMAATPADVKEYPNSYVFVVDMPGLKSGDIKVQVEEDNVLLISGERKREEEKEGAKFIRMERRVGKFMRKFSLPENANTDAISAVCQDGVLTVTVQKLPPPEPKKPKTIEVKVA* |
| > SlHSP23.8B (Solyc08g078700) MATLALRRATASSLFNRLVNPVRSASAFRSFNTNTQMTAYDQDDRGVDVDRRSDRSVSRRDAFPSLFSDVFDPFSPPIRSVSQLLNMMDQMMDSPFVAAPRAMGAGVGARRGWDVKEDDNALYIKMDMPGLDKENVKVAVEENTLIIKGEGEKESENEEYRRRYSTRLEIPQNIYKLDGIKAEMKNGVLKVAVPKVKQEERKDVFDVKIE* |
| > SlHSP21.5B (Solyc08g078710) MAFRTALRRISSSSILFYNLLNNAPSSRGLTGSLAPLTSRFLSYVGSVSVTDSNEESFIINACGGSFARFEQFPIHRGACDAYMMNPFQISGPGGAYEAKNIEEGMHVRMEMPGIDKEDVKVLISYGTIIIKGEGKKESTYEDSGRTYSANIEICSNSYEAQSMEANMKNGVLRMLIPKSKTPQKVTGSNYEIKVK* |
| > SlHSP18.2 (Solyc08g078720) MALTIRKAAGASTLFKLLSSKSKFTTAAPSITRFFSSITTADSNSLSNEQKNSTEPILVTGSPQEFKMENPFQSAGPKEVLEVDTLKDGILVRVAMPSVGEDGIKVWLENNTVYFTGKGDIEVESEESGRKYGGSLEFSTDCCKAEKVEAQMKNGILRMVIKGEMGED* |
| > SlHSP26.8 (Solyc09g007140) MDSKGAAPNQVYEDFVPTTELVQEQDSDTLLLDLTGFRKEQVRVQLTRTGVVKISGQRPVAENKWLRFQKDFPVSQNCDRTKISAKFENGILYVKQPKLITTSPQKKDQELPTSDPQQPNDEPQPTSQKKDEQQTQDEKTQTEELPKHQATNAEKPEMEEQDTKETPAEYTGASSTMEEENKPSYACKLDKDAYTRTADVVAEKLKMPRKLMNMTLIALLVLGIGLYISNKMKSNN* |
| > SlHSP24.5 (Solyc09g011710) MSLMPVFGGRRNHAHQVHDPYSDHQTHKVHDPYSDHKTHKMHDPYSRPSHPVYDPYSQHAHQVHDPFAHHDVWDPFHEFYLENPRSLIAPAPSFHHVPATMAQIEYKETPESHIFRCNLHGYKKEDVKVQVEDEKILKITGETRMMKKEDNWHHYERSSGKFFTSFSLPLNSRADYVKSSMENGVLTITIPKKEISRNHHHIRSVQIN* |
| > SlHSP15.2 (Solyc09g015000) MSLIPSFFGGRRSNIFGPFSLDLWDPFEGFPIANTPSSAREISAFANAKIDWKETPQAHVFKVDVPGIKKEEEKNDQWHRMERSSGKFKRRFRLPENAKTGEIKAEMENGVLTVTVPKEEEKKKSEVKAIDISG* |
| > SlHSP17.7B (Solyc09g015020) MSLIPSFFGGRRSNIFDPFSLDLWDPFPISTNTPYSAPEISDFANAKIDWKETPESHVFKVDVPGIKKEEVKVEVEEGRILQISGERNREKEEKNDQWHRMERSSGKFIRRFRLPENAKTGEIKAAMENGVLTVTVPKEEEKKKPEVKAIDISG* |
| > SlHSP7.8 (Solyc09g059210) MYNSAFANTRVDWKETPEAHVFKSDLPGFKKEEVKMEIEEDKVLQISGERTMEKEDKNNVNSIEISD* |
| > SlHSP15.5 (Solyc10g076880) MENKLQGHFEPTCEWQHQDGADLLLVHLPEFKDKEGLKVQVSNCGVVKISGDVQANQTRLSFLKEIQLGKDHNVDAIKANFEKGVLKITVPNKNTNEVSNKSRFKKVKKVALGVVAIVVVVSAFSGFAYYIYRSTIVKD* |
| > SlHSP27.1 (Solyc10g086680) MSLVPLFNGRKNHSRCVQNSPPTTTITPQSQKNHNRSDSYDPTTTIMHQYKPNRGNEKDLYNYPPPTRNQDLHNLFNQLATTKFTHQFQTSRGQKDPYDPSHKFYLETPRSLIAPSLSFPHVTPFLAQFECNVTPEAYVFRANNLHGYKKEEVKVQVEDDRILKISGEKKIVEKEYDNWHHFQKKVGKFSTVFNLPEDAGVDKVISTMEKEVLIVTIPKKGAVKKSHVRTVRIF* |
| > SlHSP21.5C (Solyc11g020330) MSVISKLTLLIISIACIFQVSSLSADGSSLVPLIIDQMISSNPANTFLDPFKVLEQIPFGLENTLLARVDWKETAKGHVISVEVPGLKKDDIKIEIEENRVLRVSGERKKEEEKNDEENHWHCVERSYGKFWRQFRLPENADIDTMKAKLENGVLTISFAKLSADRIKGPKVVSIESKQQGKESSVREEL* |
| > SlHSP27.5 (Solyc11g071560) MDSKGAASNQTYEDFVPTTELVQEQDSDILLIHLTGFKKEQVRVQLTKTGILKISGQRPVAESKWLRFQKDFPVSQNCDKTKISAKFENGILHVKQPKLITSSENKGQELPTSDAQQQQKPADEPQSTPQRKDEQQTKDEKTPTPTEELPKHQDTNADKPEMEEPNTKEANDLAEKTPAERTGASSTVEDGNKPSYACKLDKDAYTGTATVLAEKLKMPRKLMNMTLIALLVLGIGLYISNKMKSNN* |
| > SlHSP9.0 (Solyc12g042830) MVEDGILTIKGEHKQEKEEEGSDDEFWSSTSYGYYNNSIVLPQDAKVDEIKAEMKDGVLTITIPKSDKPKKDVKEIEVL* |
| > SlHSP27.2 (Solyc12g056560) MDTELRSKVARVSDEFNSNEFFQFPRDRAGVAFQSTETNSMFVVTAHLKGYTRGNIKVDLNENKTKLVVTCEKPVQETLTIGHEVIKKDVQIRKFTKSIQIPDGVIVDEIITNFNEETSNLTITMPKRLKEPELVTQTPCTILKRARFQEDEGSADSVTASRKGLIEQHDAKDEVPERKIETGECRNLKNDEVCEKEEDKLPKRSKVCVPVIVGSGVMLSVVVFVILFMRKKKQPGKRKA* |

**Supplementary Table 2: List of genes and their primer sequences used for quantitative real–time PCR (qRT-PCR) analysis**

| Gene Name | Gene Bank ID | SGN ID | Forward Primer (5′-3′) | Reverse Primer (5′-3′) |
| --- | --- | --- | --- | --- |
| *SlTIP41* | NM_001322431.1 | Solyc10g049850.1.1 | AACCACATTTCAGGCCTTGTCTT | CATGGAGTTTTTGAGTCTTCTGCAT |
| *SlUBI3* | X58253 | Solyc01g056940.3.1 | TCGTAAGGAGTGCCCTAATGCTGA | CAATCGCCTCCAGCCTTGTTGTAA |
| *SlHSP17.7A* | NM_001279116.2 | Solyc06g076520.1.1 | AGTTAAGGCGTCTATGGAGAATGG | AATACTACAGGTTTGTTCCCAACCA |
| *SlHSP17.7B* | XM_015231817.1 | Solyc09g015020.1.1 | TCATCGTCAAGTCATCAAGCA | TGGTTGAAATTGGGAATGGA |
| *SlACS2* | X59145.1 | Solyc01g095080.2.1 | GAGTTCGATGGAAAAGAAGCAACA | GTGACGAAAGTGGTGACAAAACAC |
| *SlMADS-RIN* | NM_001247047.2 | [Solyc05g012020.2.1](http://pgsb.helmholtz-muenchen.de/plant/tomato/reportsjsp/geneticElement.jsp?geneID=301102) | CATTGCCTATAAGTTACGGATACGA | ATTCAAAGCATCCATCCAGGTACA |

**Supplementary Table 3: List of *cis-*elements present in *SlHSP17.7A* promoter analyzed by Plant Care and PLACE promoter databases (Higo et al., 1999; Lescot *et al*., 2002)**

| ***cis-*element** | **Sequence (5′-3′)** | **Position** | **Strand** | **Function** |
| --- | --- | --- | --- | --- |
|  |  |  |  |  |
| 5UTR Py-rich stretch | TTTCTCTCTCTCTC | 976 | - | Conferring high transcription levels |
|  | TTTCTTCTCT | 981 | - |  |
| AE-box | AGAAACTT | 1314 | + | Module for light response |
| HSE | AAAAAATTTC | 134 | + | Heat stress responsiveness |
|  | AGAAAATTCG | 1474 | + |  |
|  | AGAAAATTCG | 1101 | + |  |
|  | AAAAAATTTC | 453 | - |  |
|  | AAAAAATTTC | 1117 | - |  |
| MBS | CAACTG | 149 | - | MYB binding site involved in drought-inducibility |
| SKN-1 motif | GTCAT | 1133 | - | Required for endosperm expression |
| TC-rich repeats | ATTTTCTTCA | 1098 | - | Defense and stress responsiveness |
|  | ATTTTCTCCA | 1375 | - |  |
| TCA-element | CAGAAAAGGA | 281 | - | Salicylic acid responsiveness |
| As-2-box | GATAATGATG | 967 | + | Shoot-specific expression and light responsiveness |
| chs-CMA1a | TTACTTAA | 663 | - | Light responsive element |
| CArG motif | CAAAAAAACG | 29 | - | MADS-BOX TF binding sites |
|  | CAAAAAAAAG | 1841 | - |  |
|  |  |  |  |  |

**Supplementary Table 4: List of *cis-*elements present in *SlHSP17.7B* promoter analyzed by Plant Care and PLACE promoter databases (Higo et al., 1999; Lescot *et al*., 2002)**

| ***cis-*element** | **Sequence (5′-3′)** | **Position** | **Strand** | **Function** |
| --- | --- | --- | --- | --- |
| ACE | GACACGTATG | 1152 | + | Light responsiveness |
| AE-box | AGAAACTT | 1302 | + | Module for light response |
| ARE | TGGTTT | 647 | - | Essential for the anaerobic induction |
| AT1-motif | ATTAATTTTACA | 1119 | - | Light responsive module |
| BOX4 | ATTAAT | 260 | + | Light responsiveness |
|  |  | 1125 | - |  |
|  |  | 602 | + |  |
|  |  | 1142 | - |  |
|  |  | 426 | + |  |
|  |  | 1073 | - |  |
| Box I | TTTCAAA | 937 | - | Light responsive element |
| Box III | CATTTACACT | 110 | - | Protein binding site |
| Box-W1 | TTGACC | 612 | - | Fungal elicitor responsive element |
| CATT-motif | GCATTC | 39 | - | Light responsive element |
|  |  | 328 | - |  |
| CGTCA-motif | CGTCA | 466 | - | MeJA-responsiveness |
| G-Box | CACGTT | 1002 | + | light responsiveness |
| G-box | CACGTC | 467 | - | Light responsiveness |
|  | CACGTT | 1002 | + |  |
| GARE-motif | AAACAGA | 1480 | - | Gibberellin-responsive element |
| GATA-motif | GATAGGA | 830 | - | Light responsive element |
| GCN4_motif | TGTGTCA | 896 | + | Involved in endosperm expression |
|  | TGAGTCA | 988 | + | Involved in endosperm expression |
| HSE | AAAAAATTTC | 883 | - | Heat stress responsiveness |
| I-box | GATAAGATA | 823 | - | Light responsive element |
|  | GATATGG | 954 | - |  |
| MBS | TAACTG | 969 | + | MYB binding site involved in drought-inducibility |
| MRE | AACCTAA | 652 | + | MYB binding site involved in light responsiveness |
| O2-site | GATGATGTGG | 344 | + | Zein metabolism regulation |
|  | GATGATATGG | 954 | - |  |
|  | GTTGACGTGA | 464 | + |  |
|  | GATGACATGA | 1426 | - |  |
| Skn-1_motif | GTCAT | 421 | - | Endosperm expression |
|  |  | 1430 | + |  |
|  |  | 1037 | - |  |
|  |  | 899 | + |  |
|  |  | 1112 | - |  |
| TC-rich repeats | ATTTTCTTCA | 201 | + | Defense and stress responsiveness |
|  | ATTTTCTTCA | 1335 | - |  |
|  | ATTCTCTAAC | 243 | + |  |
|  | ATTTTCTTCA | 1483 | + |  |
| TCA-element | CCATCTTTTT | 832 | + | Salicylic acid responsiveness |
|  | GAGAAGAATA | 1334 | + |  |
| TCT-motif | TCTTAC | 592 | + | Light responsive element |
|  | TCTTAC | 1309 | + |  |
| TGACG-motif | TGACG | 466 | + | MeJA-responsiveness |
| as-2-box | GATAatGATG | 1418 | - | Shoot-specific expression and light responsiveness |
| CArG motif | CTTAAATATG | 633 | - | MADS-BOX TF binding sites |
|  | CATTAATTTG | 1067 | - |  |
| circadian | CAANNNNATC | 200 | - | Involved in circadian control |
|  |  | 1435 | + |  |
|  |  |  |  |  |

**Supplementary Figure 1. Expression of *SlHSP17.7A* and *SlHSP17.7B* genes in response to ethylene (ETH) treatments to leaf tissues.** 3-4 weeks old WT (Ohio8245) plants were treated with 25 ppm ethylene. Samples were then harvested at 0, 24, 48, 72 and 96h, total RNA was isolated and expression of *SlHSP17.7A* and *SlHSP17.7B* genes was quantified. Error bars indicate standard deviation from a minimum of three replicates. *SlTIP41* and *SlUBI3* genes were used to normalize the expression of the target genes as described in methods. Statistical differences [*P≤0.05, **P≤0.01, ***P≤0.001 and ****P≤0.0001] are indicated.
